# 

**Published:** 1882-04

**Authors:** 


					﻿Whole Number,
<_CCCCXLVII.
April, 1882.
Number 4,
Vol. XLIV.
THE
CHICAGO
M E me AB
T ournal and Examiner
EDITORS:
N. S. DAVIS. M.D., LL.D. JAS. NEVINS HYDE, A.M., M.D.
DANIEL R. BROWER, A.M., M.D.
CHICAGO:
PUBLISHED BY THE CHICAGO MEDICAL PRESS ASSOCIATION,
188 Clark Street.
^g*Read the Notice of this Journal among Advertisements
				

